# Immune responses of human T lymphocytes to novel hepatitis B virus-derived peptides

**DOI:** 10.1371/journal.pone.0198264

**Published:** 2018-06-01

**Authors:** Daisuke Yamamiya, Eishiro Mizukoshi, Kiichiro Kaji, Takeshi Terashima, Masaaki Kitahara, Tatsuya Yamashita, Kuniaki Arai, Kazumi Fushimi, Masao Honda, Shuichi Kaneko

**Affiliations:** Department of Gastroenterology, Graduate School of Medicine, Kanazawa University, Kanazawa, Ishikawa, Japan; Academia Sinica, TAIWAN

## Abstract

**Background & aims:**

Many individuals are infected with hepatitis B virus (HBV) worldwide, and this virus is commonly controlled by treatments with interferon (IFN)-alpha and nucleoside analogues (NA). However, the complete elimination of HBV by these treatments is difficult and, thus, the development of new treatments is needed. Host immune responses are closely involved in the elimination of HBV, suggesting the usefulness of immunotherapy. In the present study, we attempted to identify novel cytotoxic T-lymphocyte (CTL) epitopes that are useful for immunotherapy against HBV.

**Methods:**

CTL epitopes were predicted using computer software. Immune responses to each peptide were evaluated by IFN-γ ELISPOT and cytotoxic assays. The relationships between the immune responses to these newly identified CTL epitopes and the clinical backgrounds of patients and administration of NA were analyzed. Peptides were administered to mice as vaccines and peptide-specific T-cell induction was measured *in vivo*.

**Results:**

Positive reactions to 10 synthesized peptides were detected in 3 or more patients using the IFN-γ ELISPOT assay, and concentration-dependent cytotoxicity against 2 of these peptides was observed in the cytotoxic assay. Some peptides that correlated with serum ALT, HBsAg, and HBV core-related antigen (HBcrAg) levels were identified. Immune reactions against some peptides were enhanced by the administration of NA. Regarding their effects as a vaccine, peptide-specific T-cells were induced by four peptides *in vivo*.

**Conclusions:**

Novel HBV epitopes that correlated with HBsAg and HBcrAg levels were identified. These newly identified epitopes may be useful in the analysis of immune responses to HBV and development of immunotherapy against HBV.

## Introduction

Interferon (IFN)-α and nucleoside analogues (NA) are used in the treatment of hepatitis B virus (HBV), and are beneficial for many HBV-infected patients [1,2]. Treatments with NA inhibit the reverse transcription activity of HBV polymerase, which rapidly suppresses viral replication and attenuates hepatitis in many patients. However, HBsAg is not eliminated and the risk of recurrence is high when drug administration is discontinued or an immunosuppressive state develops [3]. This is due to the difficulties associated with the complete elimination of covalently closed circular DNA (cccDNA) by current treatments. In order to eradicate HBV infection, it is important to develop a new treatment method that eliminates cccDNA.

Advances have been achieved in immunotherapy over the past several years, and the efficacy of immune checkpoint inhibitors, such as anti-PD-1, anti-PD-L1, and anti-CTLA-4 antibodies, against various malignant tumors has been reported in actual clinical practice and clinical studies [4]. Regarding HBV, anti-HBs antibody-positive individuals are considered to have recovered, with reactivation being rare. A prospective study involving HBV patients also suggested that HBV is controlled by immune responses [5]. This is also indicated by the incidence of recurrence after the withdrawal of IFN, which not only eliminates viruses, but also has immunomodulatory functions, being lower than that after the withdrawal of NA and also the rate of HBsAg seroconversion induced by IFN being higher [6]. Therefore, in order to expel cccDNA, it is important to completely remove infected hepatocytes, for which immunotherapy is likely to be effective.

The close involvement of the strength of immune reactions, particularly cytotoxic T lymphocytes (CTLs), in whether HBV is eliminated or chronically infects has been reported [7,8], and the virus-eliminating action of cytokines produced by CTLs, such as IFN-γ, has been demonstrated in an animal study using chimpanzees [9]. CTLs are one of the cell types serving as the center of immunity eliminating viruses: they recognize endogenously synthesized complexes of MHC class I molecules and viral epitopes presented on the surface of infected cells and exhibit cytotoxicity. The involvement of many epitopes in the clearance of HBV in acute and chronic hepatitis has been reported, and the immune reaction of CTLs is strong in acute hepatitis patients and recovered patients, but weak in chronic hepatitis patients [10–13].

Therefore, host immune reactions are important for completely eliminating HBV and many immunotherapies against HBV have been investigated, but have yet to be established. Several HBV-derived CTL epitopes have been identified [14,15]; however, the identification of highly immunogenic CTL epitopes is necessary for inducing strong immune responses. HBV-DNA, HBV core-related antigen (HBcrAg), and HBsAg quantification methods have recently been developed [16] and have facilitated the accurate analysis of CTL epitopes associated with the state of HBV in the body.

In the present study, in order to identify highly immunogenic novel T-cell epitopes derived from HBV, we narrowed down candidate epitopes using various immunological methods and closely analyzed the immune responses of HBV-infected patients to these epitopes.

## Patients and methods

### Patients

We examined 62 HLA-A24-positive patients with previous or present HBV infection. All subjects were negative for Abs to human immunodeficiency virus (HIV) and hepatitis C virus (HCV). They were grouped as follows: group 1, 9 patients with acute hepatitis; group 2, 12 chronically infected patients who were positive for HBsAg and not being treated with NA; group 3, 32 chronically infected patients who were positive for HBsAg and being treated with NA; group 4, 3 recovered patients who were negative for HBsAg and positive for HBsAb; and group 5, 6 HBV carriers who were positive for HBsAg, but have sustainable normal ALT levels (≤30 U/L) and were not being treated with NA. Groups 2 and 3 were subdivided into patients without (groups 2a and 3a, respectively) or with HBeAg in their sera (groups 2b and 3b, respectively). NA-induced changes in immune responses were examined using 12 patients in group 2. The administration of NA was part of routine clinical care. All patients gave written informed consent to participate in this study in accordance with the Helsinki declaration, reflected in *a priori* approval by the regional Ethics Committee (Medical Ethics Committee of Kanazawa University, No. 1259).

### Laboratory and virologic testing

Serum HBsAg, anti-HBs, HBeAg, anti-HBe, and HBcrAg levels were tested with commercial immunoassays (Fuji Rebio, Tokyo, Japan). The HBV genotype was also tested with commercial immunoassays (SRL, Tokyo, Japan). The HLA-based typing of peripheral blood mononuclear cells (PBMCs) from patients was performed by reverse sequence-specific oligonucleotide PCR using LABType SSO (One Lambda, Canoga Park, CA). Serum HBV DNA was tested by real-time PCR with a lower limit of detection of 2.1 log copies per milliliter (Roche Diagnostics, Indianapolis, IN).

### Peptides and cell lines

MHC class I-restricted peptides were synthesized at Eurofins (Tokyo, Japan) at >90% purity. The HLA-A*2402 gene-transfected C1R cell line (C1R-A24) was cultured in RPMI 1640 medium containing 10% fetal calf serum (FCS) and 500 μg/mL hygromycin B (Sigma, St. Louis, MO), and K562 was cultured in RPMI 1640 medium containing 10% FCS. C1R-A24 and K562 was kindly provided by Dr. Takiguchi (Center for AIDS Research, Kumamoto University, Kumamoto, Japan). HepG2 (human hepatoma cell line) was cultured in DMEM (Gibco, Grand Island, NY) with 10% FCS. HepG2 was provided by the RIKEN BRC (RCB1886).

### IFN-γ ELISPOT assay

The IFN-γ ELISPOT assay was performed as previously described with duplicate cultures of 3 × 10^5^ PBMCs [17]. Negative controls consisted of an HIV envelope-derived peptide (HIVenv_584_) [18]. Positive controls consisted of 10 ng/mL phorbol 12-myristate 13-acetate (PMA, Sigma) or a cytomegalovirus (CMV) pp65-derived peptide (CMVpp65_328_) [19]. The number of spots was counted with a KS ELISPOT Reader (Zeiss, Tokyo, Japan). A response was scored as positive if more than 10 specific spots (number of spots in the presence of the HBV antigen minus that in its absence) were detected and if the number of spots in the presence of the HBV antigen was at least two-fold greater than the number in its absence.

### Peptide binding assay

The peptide-HLA binding assay was performed as previously described [20]. T2-A24 cells (transporter associated with antigen processing [TAP]-deficient human lymphoid-derived cells transfected with the HLA-A*2402 molecule) were cultured at 26°C for 16 hr. After the addition of synthetic peptides, they were incubated with the peptides at 37°C for 2 hr. Cells were then stained with a FITC-conjugated mouse anti-HLA-A24 monoclonal antibody (MBL, Nagoya, Japan) and 1 μg/mL of propidium iodide (PI). Live cells were gated based on forward and side scattering and the exclusion of PI-positive cells. Data was expressed as a % Mean Fluorescence Intensity (MFI) increase, which was calculated as follows: % MFI increase = (MFI with the given peptide—MFI without the peptide) /(MFI without the peptide) × 100.

### CTL induction

CTLs were expanded from PBMCs in 96-well round-bottomed plates as previously described [21]. Briefly, 400,000 cells/well were stimulated with synthetic peptides at 10 μg/mL, 10 ng/mL rIL-7, and 100 pg/mL rIL-12 (Sigma, St. Louis, MO) in RPMI 1640 supplemented with 10% heat inactivated human AB serum, 100 U/mL penicillin, and 100 μg/mL streptomycin. Cultures were re-stimulated with 10 μg/mL peptides, 20 U/mL rIL-2 (Shionogi, Osaka, Japan), and 10^5^ mitomycin C-treated autologous PBMCs on days 7 and 14. On days 3, 10, and 17, 100 μL of RPMI with 10% human AB serum and 10 U/mL rIL-2 was exchanged in each well.

### Cytotoxicity assay

A cytotoxicity assay was performed using a chromium-release assay as previously described [20]. C1R-A24 cells were used as target cells. C1R-A24 cells were incubated overnight with 10 μg/mL synthetic peptides and labeled with 25 μCi ^51^Cr (PerkinElmer, Waltham, MA) at 37°C for 1 hr. After washing, target cells were plated at 3,000 cells/well with complete medium in 96-well round-bottomed plates. Unlabeled K562 cells at 120,000 cells/well were added to reduce non-specific lysis. Stimulated PBMCs were added at effector to target ratios of 50:1, 25:1, 12.5:1, and 6:1. Chromium release was measured in the supernatant after a 4-hr incubation at 37°C and maximum release was assessed by the lysis of ^51^Cr-labeled targets with 5% Triton X-100 (Sigma, St. Louis, MO). Percent cytotoxicity was evaluated from the formula: 100 × [(experimental release—spontaneous release) / (maximum release—spontaneous release)]. Specific cytotoxic activity was calculated as follows: (cytotoxicity in the presence of the peptide)—(cytotoxicity in the absence of the peptide). Spontaneous release was <15% of the maximum release for all experiments. As a negative control, we used an HIV envelope-derived peptide (HIVenv_584_) [18].

### Immunization of mice

Six- to seven-week-old male HLA-A24 transgenic mice were obtained from SLC (Shizuoka, Japan). The mice were kept on a 12-h light/dark cycle with free access to food and water. Two weeks after obtaining, mice were immunized with 120 μg of the peptide on days 0, 7, and 14. Peptides were emulsified in Montanide-ISA-51 with saline. Only Montanide-ISA51 with saline was injected as a control. Mice were sacrificed 1 week after the last immunization and splenocytes were dispersed for the ELISPOT assay. The mice were randomly assigned and each group contained three mice. All experiments involving mice were conducted in accordance with institutional guidelines and were prospectively approved by the Ethics Committee (The Kanazawa University Animal Experiment Committee, No.142986). Intraperitoneal pentobarbital overdose was performed as the procedure for euthanasia.

### HBV transfection

A strain of HepG2 cells engineered to overexpress the human NTCP gene (HepG2-NTCP-C4 cells) [22] was used. HepG2-NTCP-C4 cells were transfected with a pUC19 vector carrying 1.24-fold the HBV genome of genotype C (pUC19-HBV-C) [23] using the Fugene 6 transfection reagent (Roche Diagnostics, Indianapolis, IN). HepG2-NTCP-C4 was kindly provided by Dr. Watashi (National Institute of Infectious Diseases, Tokyo, Japan). pUC19-HBV-C was kindly provided by Dr. Tanaka (Nagoya City University, Nagoya, Japan).

### Statistical analysis

The Student’s *t*-test (two-tailed) was used to compare Age, ALT, HBsAg, HBeAg, HBcrAg, and HBV-DNA levels in two groups. The Fisher’s exact test was used to compare positive rate of HBeAg. A value of p < 0.05 was considered to be significant.

## Results

### Patient profiles

The clinical background factors of the patients analyzed are shown in Table 1. There were 9 patients with acute hepatitis (group 1), 12 with chronic hepatitis before the administration of NA (group 2) (HBeAg-negative: 8, positive: 4), 32 with chronic hepatitis after the administration of NA (group 3) (HBeAg-negative: 28, positive: 4), 3 recovered patients positive for the HBs antibody (group 4), and 6 carriers (group 5). Median ages were 41, 49, 61, 66, and 58 years in groups 1, 2, 3, 4, and 5, respectively. The complication of hepatic cirrhosis was noted in the background liver in 8 and 7 patients in groups 2 and 3, respectively. The HBV genotype was B in 2, 1, 3, 0, and 1 patient in groups 1–5, respectively, and none of the patients were infected with genotype A HBV. It was not possible to judge the genotype in 3, 0, 1, 1, and 1 patient in groups 1–5, respectively. All other patients were infected with HBV genotype C.

**Table 1:** Patient characteristics.

Group	Clinical Diagnosis	No.	Sex M/F	Age(yr) Mean ± SD	No. of CH/LC	Genotype (A/B/C/ND)	ALT(IU/L) Mean ± SD	HBsAg(IU/ml) Mean ± SD	HBV DNA (log copies/ml) Mean ± SD	HBcrAg* (log U/ml) Mean ± SD
1	Acute hepatitis	9	4/5	41±13	-	0/2/4/3	1177±1454	501±768	5.5±2.0	4.5±1.6
2	Chronic hepatitis, no therapy									
2a	HBeAg negative	8	6/2	49±9	1/7	0/1/7/0	46±36	1648±471	4.7±1.5	4.7±0.9
2b	HBeAg positive	4	4/0	49±10	3/1	0/0/4/0	319±499	10175±12047	8.3±1.0	6.6±0.6
3	Chronic hepatitis, on therapy									
3a	HBeAg negative	28	20/8	61±10	22/6	0/3/24/1	29±38	1462±2682	1.5±1.0	3.8±0.7
3b	HBeAg positive	4	2/2	61±14	3/1	0/0/4/0	34±22	4175±2598	2.3±0.3	6.2±0.5
4	Recovered patient	3	2/1	66±20	‒	0/0/2/1	15±4	ND	0.7±1.2	2.9
5	HBV carrier	6	3/3	58±13	‒	0/1/4/1	24±11	8863±15423	4.3±2.5	3.6±1.5

HBV, hepatitis B virus; M, male; F, female; SD, standard deviation; CH, chronic hepatitis; LC, liver cirrhosis; ND, not determined; HBcrAg, hepatitis B core-related antigen

* <2.9 log U/ml is calculated as 2.9 log U/ml.

### Selection of potential HLA-A*2402-restricted peptides within HBV genotype C

In order to identify epitopes of HBV-derived HLA-A*2402-restricted CTL, epitopes were selected based on the binding rates predicted by computer software (BIMAS) from the amino acid sequences of the HBV genotype C large S, pre-core/core, HBx, and polymerase regions. When epitopes with a BIMAS score exceeding 5 were selected, 28, 13, 4, and 44 epitopes were extracted from the large S, pre-core/core, HBx, and polymerase regions, respectively (89 epitopes in total) (S1 Table). By including the 4 peptides previously reported as CTL epitopes, a total of 93 peptides were prepared.

### T-cell responses to HBV-derived peptides

**Fig 1:**
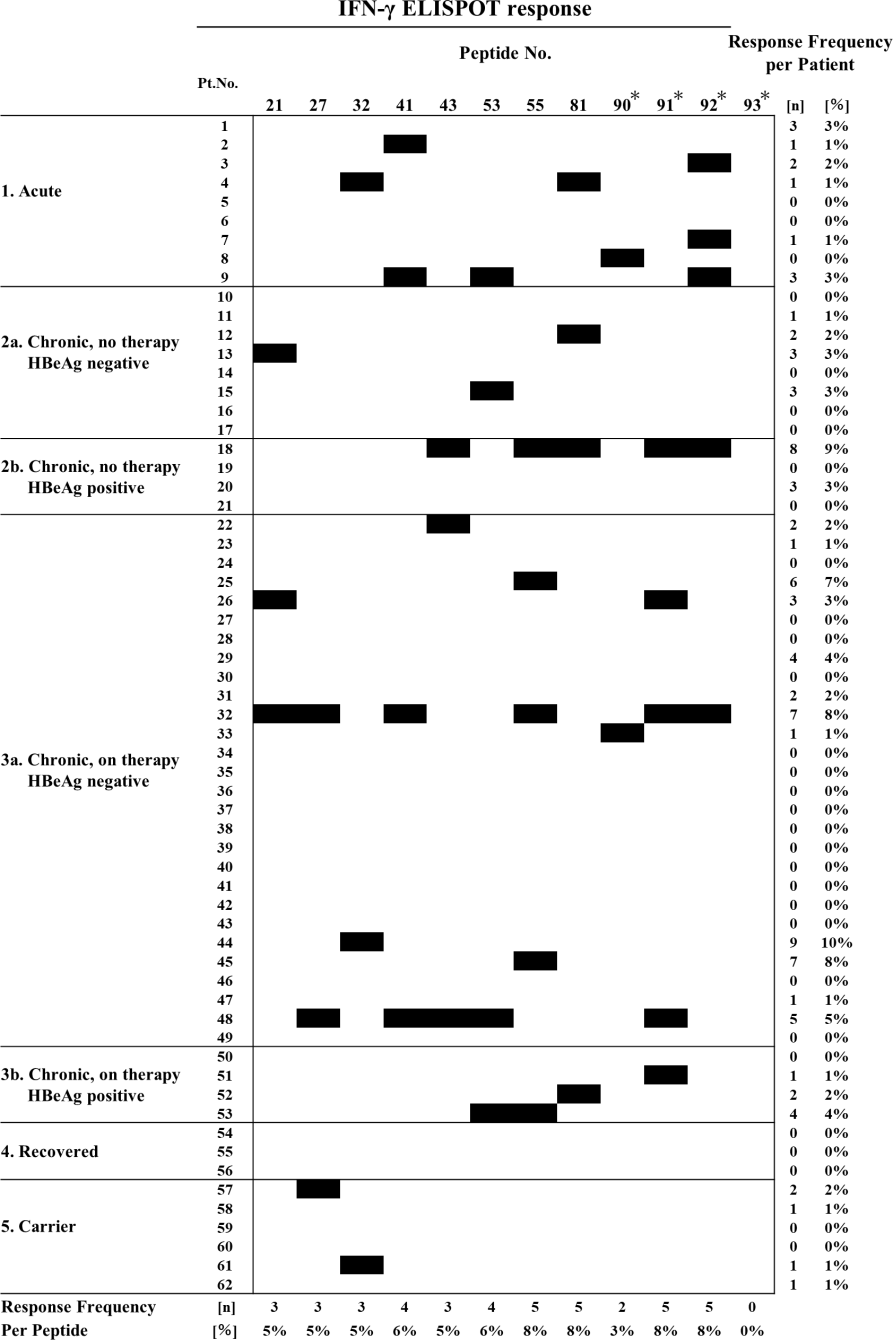
HBs, HBc, HBx, and polymerase peptide-specific CTL responses. The results of all patients are shown. The results of only twelve peptides, 4 peptides previously reported and peptides for which more than three patients were positive in the IFN-γ ELISPOT assay, are shown. Black boxes indicate a positive immune response. The asterisks are peptides previously reported.

In order to evaluate whether these peptides are recognized by the T cells of HBV patients, an IFN-γ ELISPOT assay was performed. The 93 peptides were evaluated using PBMCs collected from the 62 HBV patients described above. Ten of these peptides induced immune reactions in 3 or more patients (Fig 1). A positive reaction to the positive control, CMVpp65_328_, was detected in 20 patients (32%), whereas a positive reaction to the negative control, HIVenv584, was not detected in any patient (S1 Fig). Positive reactions to peptides 41, 53, 55, 81, 91, and 92 were detected in 4 or more patients. The numbers of spots detected in each of the 62 patients are shown in S2 Fig. Immune reactions to these peptides were more frequently detected in acute and chronic hepatitis patients, while no reaction was detected in anti-HBs antibody-positive recovered patients. In the carriers, a positive reaction to peptide 81 was detected in one patient only. The frequency of each peptide-specific T cell in peripheral blood in each pathology ranged between 13 and 152 cells/3 × 10^5^ PBMCs in acute hepatitis, 10–87 cells/3 × 10^5^ PBMCs in chronic hepatitis, and 10 cells/3 × 10^5^ PBMCs in one carrier, thereby confirming a strong immune reaction in acute hepatitis patients, as previously reported [11,17,24]. In addition, the frequencies of T cells specific for peptides 41, 53, 55, 81, 91, and 92 were 17–152, 11–33, 19–40, 10–14, 11–45, and 17–87 cells/3 × 10^5^ PBMCs, respectively. We similarly performed the IFN-γ ELISPOT assay with the 93 peptides in 17 healthy HLA-A24-positive subjects and none of the peptides induced a reaction in 2 or more subjects (S3 Fig).

### Binding of HBV-derived peptides to HLA-A*2402

**Fig 2:**
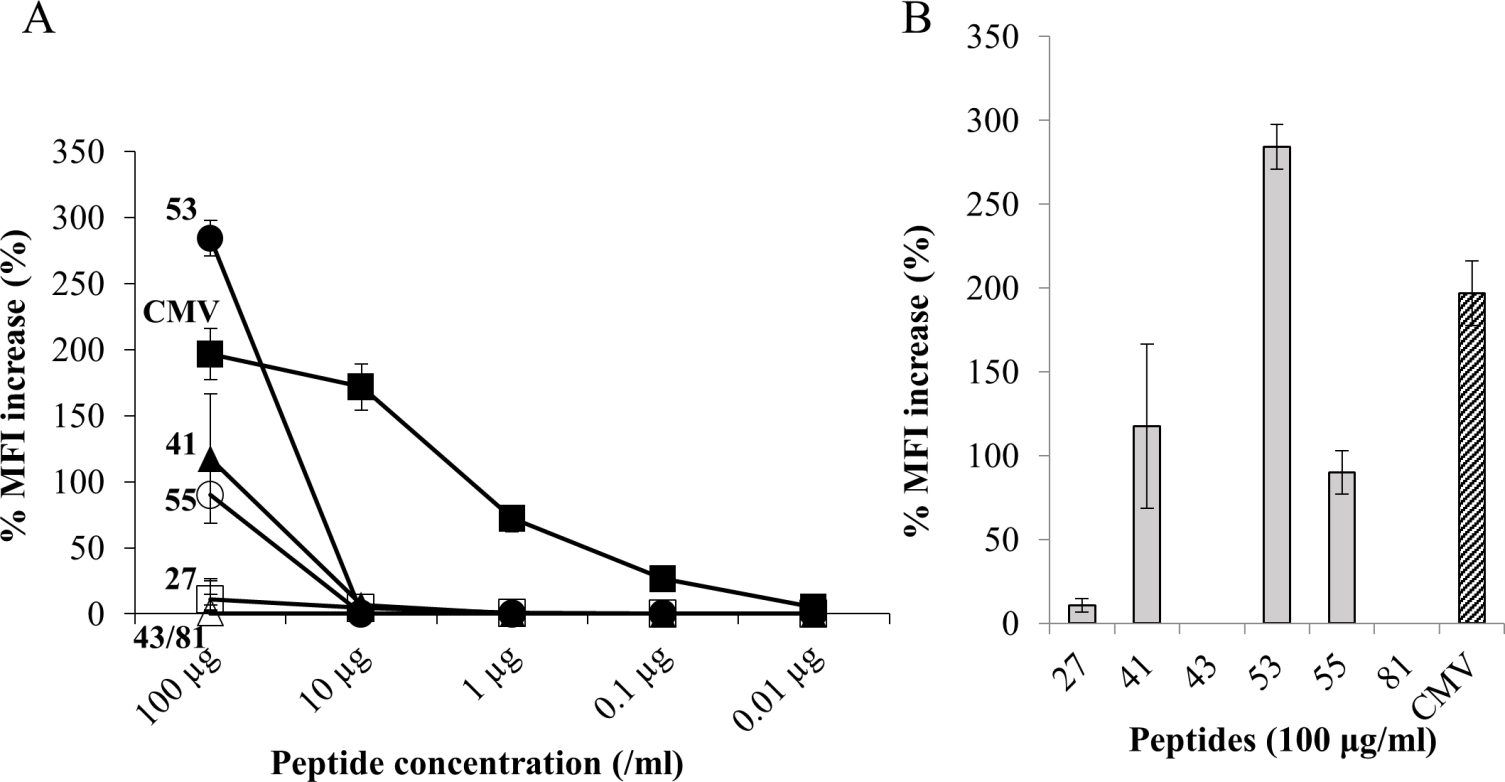
MHC binding affinity. TAP-deficient T2-A24 cells, which express the HLA-A24 molecule, were used to assess binding affinity against peptides. (A) The MHC binding affinity of 6 representative peptides and CMVpp65_328_ are shown at various concentrations. Data are expressed as the %MFI increase for live PI-negative cells. (B) The MHC binding affinities of 6 representative peptides and CMVpp65_328_ at a peptide concentration of 100 μg/mL are shown. Data was shown as mean ± SD. Abbreviations: MFI, mean fluorescence intensity; CMV, cytomegalovirus.

All the peptides designed were predicted to bind to HLA-A*2402 using a computer. In order to investigate actual affinity to the HLA-A24 molecule, peptide binding assays were performed using T2-A24-cells. Peptides 41, 53, 55, and 81, which were ELISPOT-positive in 4 or more patients, and peptides 27 and 43 (6 peptides in total) were analyzed. CMVpp65_328_ was included as the positive control. The concentration-dependent binding of CMVpp65_328_ was observed (Fig 2A). Peptides 41, 53, and 55 only bound when the peptide concentration was 100 μg/mL, and the binding of peptide 53 was stronger than that of CMVpp65_328_. In contrast, the binding of peptides 27, 43, and 81 to HLA-A24 was weak (Fig 2B).

### Cytotoxic activity of HBV-derived peptide-specific CTLs

**Fig 3:**
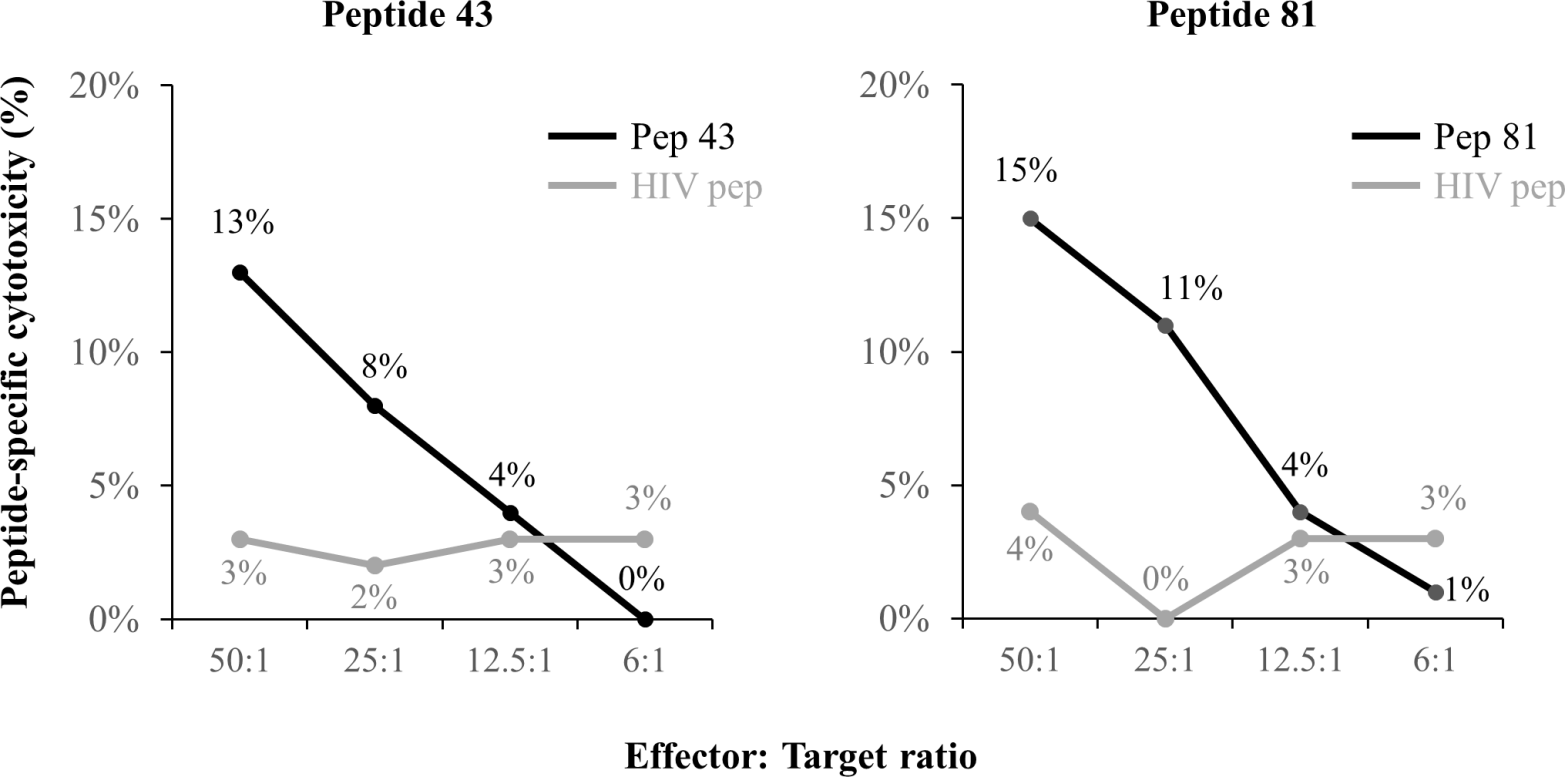
Cytotoxicity of peptide-specific CTLs in HBV patients. The cytotoxicity of CTLs was assessed at various effector to target (E/T) ratios against C1R-A24 cells pulsed with peptides. Two peptides (peptides 43 and 81), for which specific cytotoxicity was detected, are shown. Specific cytotoxicity was calculated as follows: (cytotoxicity in the presence of the peptide)—(cytotoxicity in the absence of the peptide). The black line shows peptide 43 and 81. The gray lines shows HIV peptide as a negative control. Abbreviations: pep, peptide; HIV, human immunodeficiency virus.

Peptides capable of inducing specific functional CTLs were searched for among these peptides using the PBMCs of HLA-A*2402-positive patients. Fresh PBMCs from HLA-A*2402-positive patients were stimulated with each peptide for 21 days, and cytotoxicity for C1R-A24 pulsed with the same peptide was measured. We performed a cytotoxicity assay using 10 peptides, positive reactions for which were detected in 3 or more patients by the IFN-γ ELISPOT assay. Specific CTLs induced by peptides 43 and 81 exhibited cytotoxicity to C1R-A24 (10% or higher cytotoxicity at 50:1 was detected in 2 patients each), and these reactions were concentration-dependent (Fig 3). No cytotoxicity to K562 expressing no HLA was observed. These results demonstrated that peptides 43 and 81 induced HLA-A*2402-restricted cytotoxic CTLs. On the other hand, no specific cytotoxicity was detected for peptides other than peptides 43 and 81.

### Endogenous expression of HBV-derived peptides

**Fig 4:**
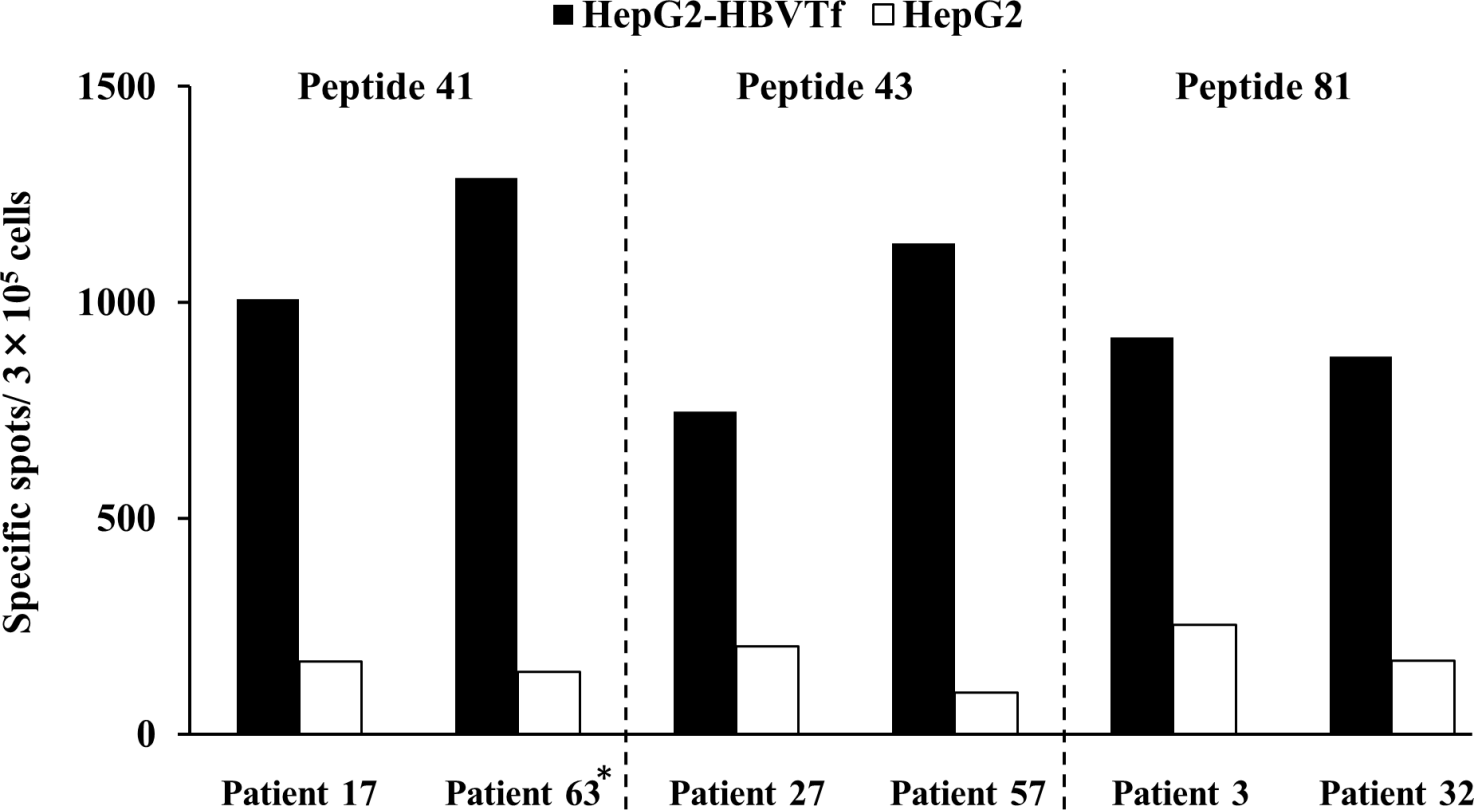
Endogenous expression of HBV-derived peptides. CTLs induced by peptide 41, 43 or 81 were incubated with HepG2-NTCP-C4 cells (HLA-A24 positive) transfected with HBV genotype C. The IFN-γ ELISPOT assay was performed in mixed wells. Black bars indicate the number of specific spots with HepG2-NTCP-C4 cells (HLA-A24 positive) transfected with HBV genotype C, and white bars indicate the number of specific spots with HepG2. The asterisk is a chronic hepatitis patient (HLA-A24-positive) transfected with HBV genotype C other than patient list (Table 1). Abbreviation: Tf, transfection.

We investigated whether peptides 41, 43, and 81 are endogenous epitopes presented by HBV-infected cells. The HBV genotype C-infected HLA-A*2402-positive cell line HepG2 was mixed with specific CTLs induced with the peptides and subjected to the IFN-γ ELISPOT assay performed in 2 patients each (Fig 4). Strong immune reactions against HBV-infected cells were observed, confirming that peptides 41, 43, and 81 are endogenous CTL epitopes of HBV.

### Peptide-specific T-cell responses and clinical features of HBV patients

**Fig 5:**
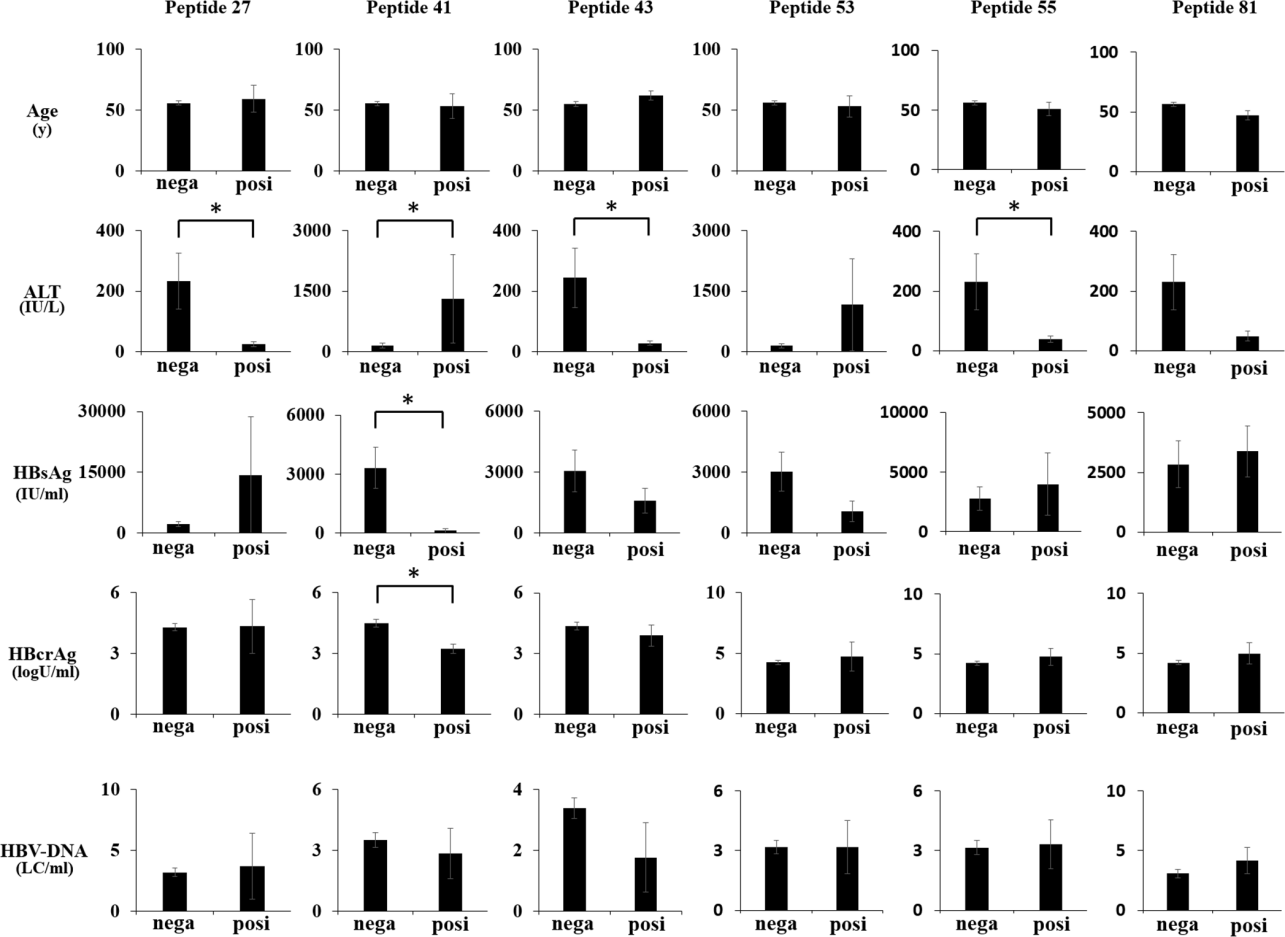
Peptide-specific T-cell responses and clinical features of HBV patients. Average ages and ALT, HBsAg, HBcrAg, and HBV-DNA levels were compared between patients who tested negative in the IFN-γ ELISPOT assay and positive for the 6 representative peptides. Data was shown as mean ± SD. Asterisks indicate p < 0.05. Abbreviations: y, years old; IU, international unit; log U, log unit; LC, log copies; nega, negative; posi, positive.

In order to investigate the characteristics of immune responses to HBV-derived MHC class I-restricted epitopes in HBV-infected patients, clinical backgrounds (age and serum ALT, HBsAg, HBcrAg, and HBV-DNA levels) were compared between patients positive and negative for immune reactions in the IFN-γ ELISPOT assay with peptides 27, 41, 43, 53, 55, and 81 (Fig 5). ALT levels were significantly higher (156±403 vs. 1,309±2,193 IU/L), while HBsAg (3,314±124 vs. 124±218 IU/mL) and HBcrAg (4.5±1.4 vs. 3.2±0.4 log U/mL) levels were significantly lower in 4 patient sera in which an immune response to peptide 41 was detected, suggesting that peptide 41 is involved in immune responses. In contrast, ALT levels were significantly lower in patients in which immune responses to peptides 27, 43, and 55 were detected (233±701 vs. 25±14, 244±719 vs. 29±20, and 231±702 vs. 39±24, respectively). No significant differences were observed in age or HBV-DNA levels in association with any peptide.

### Amino acid mutations in peptide sequences

The degree of conservation of the amino acid sequence of the epitope region in HBV-infected patients was investigated in those positive and negative for immune responses. We used the sera of 3 HBV genotype C-infected patients each positive for peptides 41 and 81 and 3 patients each negative for these peptides in the IFN-γ ELISPOT assay. As shown in Table 2, amino acid mutations were frequently detected in peptides 41 and 81 in patients with negative immune responses.

**Table 2:** Amino acid mutations in peptide sequences.

Peptide 41		L	A	T	W	V	G	S	N	L	-
Immune response	Patient No.										Amino Acid Mutation No.
**Yes**	2	***P***	***G***	T	***R***	V	G	S	N	L	3
	32	***P***	***G***	T	W	V	G	S	N	L	2
	48	L	A	T	W	V	G	S	N	***W***	1
**No**	20	***P***	***G***	T	W	V	G	***R***	N	L	3
	33	***P***	A	T	***R***	V	G	***R***	N	L	3
	49	***S***	***G***	T	***R***	***G***	G	***R***	N	L	5
Peptide 81		Q	Y	V	G	P	L	T	V	N	-
Immune response	Patient No.										Amino Acid Mutation No.
**Yes**	18	Q	Y	V	G	P	L	T	V	N	0
	25	Q	Y	V	G	P	L	T	V	N	0
	52	Q	Y	V	G	P	L	T	V	N	0
**No**	20	***H***	Y	V	***A***	P	L	***P***	V	N	3
	33	***L***	***S***	V	G	P	L	T	V	N	2
	49	Q	Y	V	G	P	L	***P***	V	N	1

### Enhancement of peptide-specific T-cell responses under NA therapy

**Fig 6:**
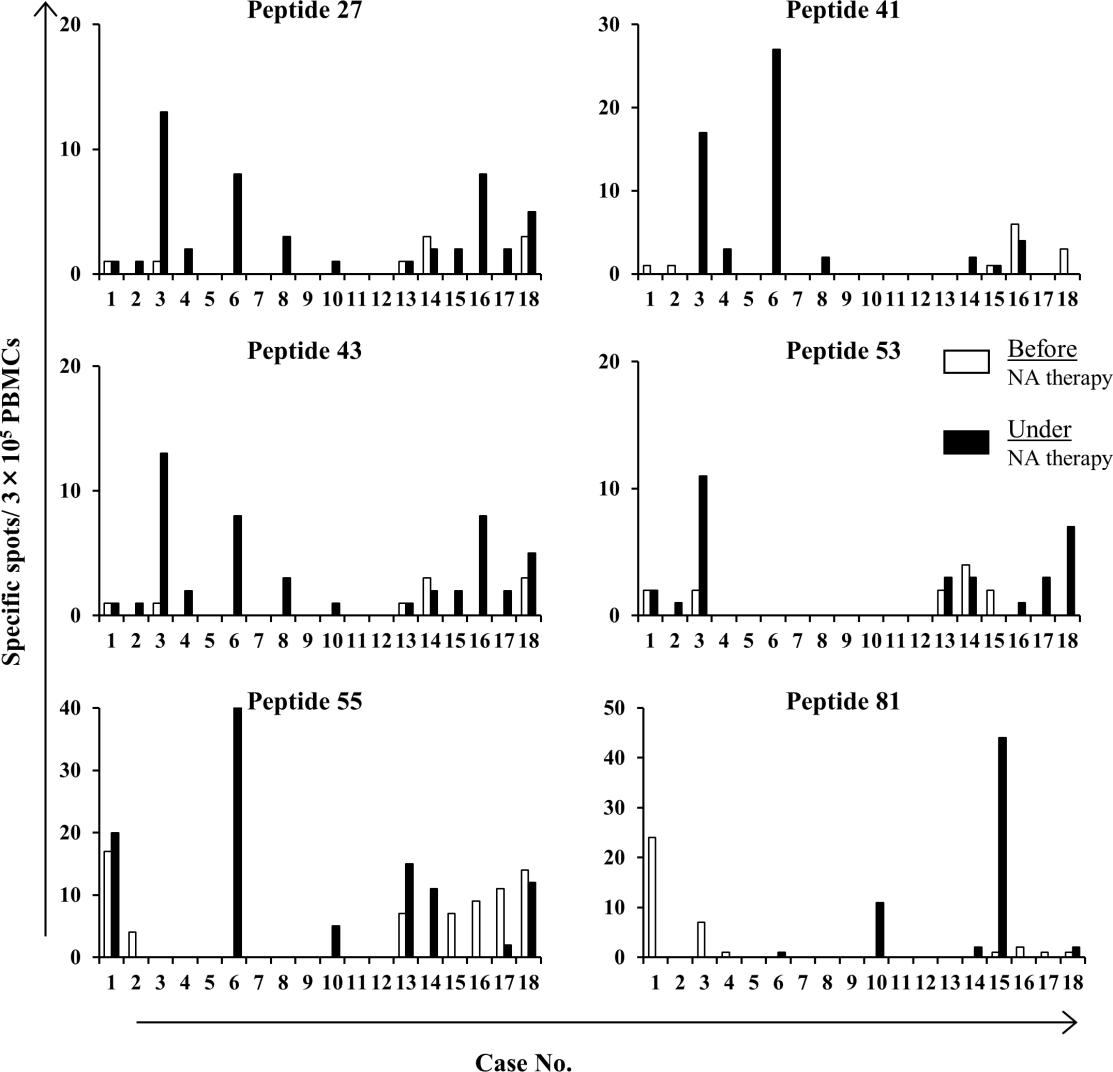
Enhancement of peptide-specific CTL responses under nucleoside analogue (NA) therapy. IFN-γ ELSIPOT assays were performed in 18 chronic hepatitis patients before and under NA therapy. White and black bars indicate the results of assays of patients before and under NA therapy, respectively. Abbreviations: PBMCs, peripheral blood mononuclear cells; NA, nucleoside analogue.

Previous studies reported that immune responses were strengthened by the administration of NA to HBV patients [17,25,26]. NA-induced changes in immune responses to these peptides were investigated using PBMCs collected before and after the administration of NA to 18 chronic hepatitis patients. The clinical backgrounds of patients before and after the treatment with NA are shown in Table 3. HBV-DNA, HBcrAg, and ALT levels were significantly decreased by the administration of NA, whereas no significant decrease was noted in HBsAg levels, as previously reported. Using PBMCs from these patients, the number of peptide-specific CTLs induced was measured using the IFN-γ ELISPOT assay. As shown in Fig 6, immune responses to these peptides tended to be enhanced after the treatment.

**Table 3:** Characteristics before/under nucleoside analogue therapy.

	No.of Patients	Sex M/F	No. CH/LC	HBeAg (+/-)	HBeAg (S/CO) Mean ± SD	Age(yr) Mean ± SD	HBsAg (IU/ml) Mean ± SD	HBV DNA (log copies/ml) Mean ± SD	HBcrAg (log U/ml) Mean ± SD	ALT(IU/L) Mean ± SD
Before therapy	18	12/6	16/2	10/8	389±613 *	54±9	5156±11113	7.2±1.3	6.1±1.5	142±217
Under therapy				3/15	71±256 *	60±9	2371±3421	1.4±1.2	4.0±1.3	24±11
p-value	ND	ND	ND	**0.035**	0.06	ND	ND	**<0.005**	**0.007**	**0.005**

M, male; F, female; CH, chronic hepatitis; LC, liver cirrhosis; SD, standard deviation; ND, not determined.

* <0.1 S/CO is calculated as 0.1 S/CO and >1600 S/CO is calculated as 1600 S/CO.

### HBV-specific CTLs *in vivo*

**Fig 7:**
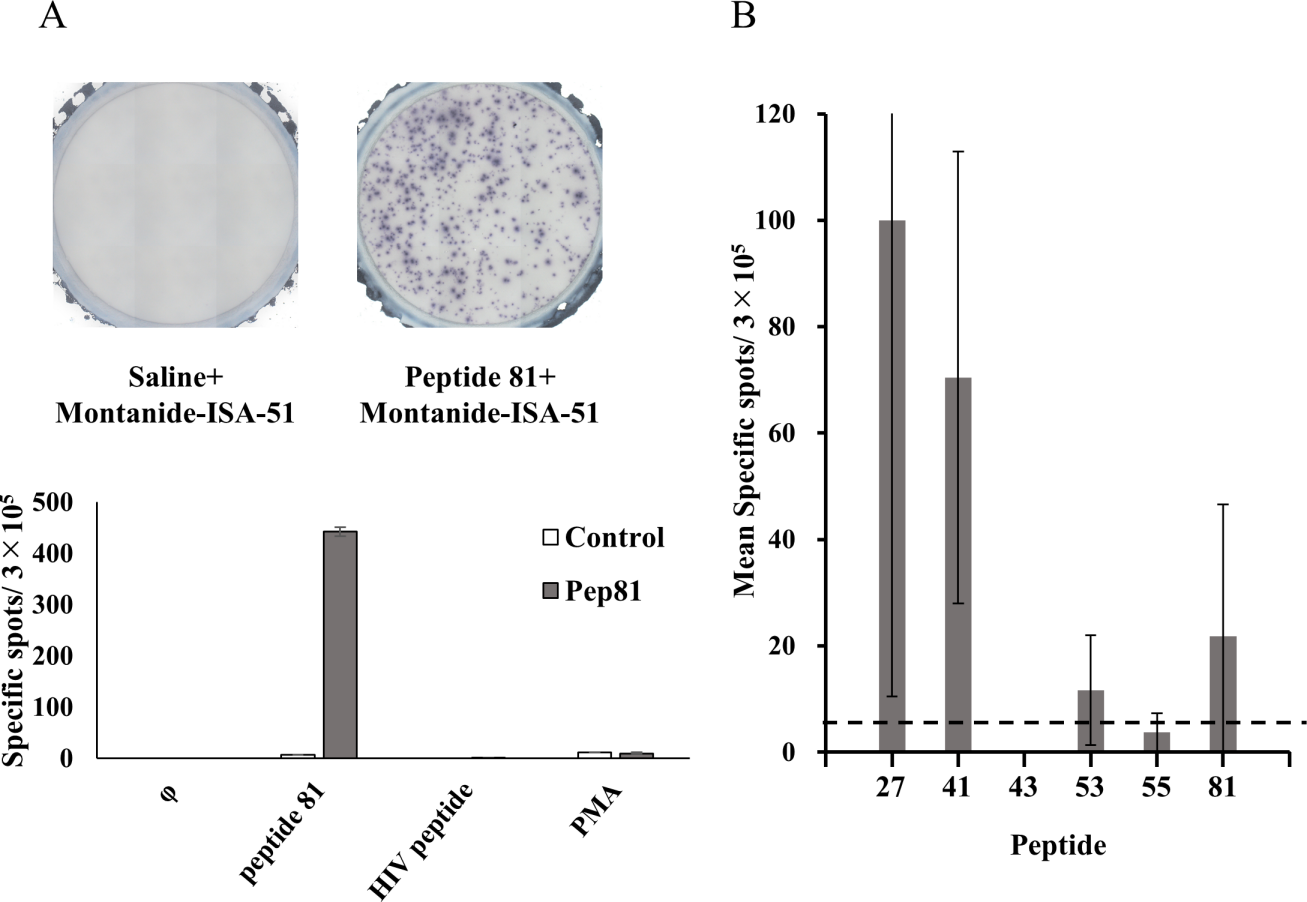
HBV-specific CTLs induced *in vivo*. HLA-A24 transgenic mice were immunized with each peptide. Peptides were emulsified in Montanide-ISA-51 with saline. The splenocytes of immunized mice were used for the IFN-γ ELISPOT assay. (A) Representative results of the assay for peptide 81 are shown. The negative control consisted of HIVenv_584_ and the positive control of Phorbol 12-myristate 13-acetate (PMA). (B) Mice were immunized with 6 representative peptides and the splenocytes of immunized mice were used for the IFN-γ ELISPOT assay. Responses were scored as positive in 4 out of 6 peptides. Data was shown as mean ± SD. Abbreviations: pep, peptide; φ, control; PMA, phorbol 12-myristate 13-acetate.

The induction of CTLs by peptides containing the epitopes identified *in* vivo was investigated. Six peptides (27, 41, 43, 53, 55, and 81) were administered to HLA-A*2402 transgenic mice. Just before the first injection(day0), the average weight of mice was 23.8g (21.0–26.5). Each peptide was subcutaneously injected into the inguinal region with Montanide-ISA51 twice (only Montanide-ISA51 was injected into the control), lymphocytes were extracted from the mouse spleen, and the IFN-γ ELISPOT assay was performed with the peptide. The lymphocytes of mice treated with peptide 81 showed a strong immune reaction (Fig 7A), and *in vivo* CTL induction by peptides 27, 41, and 53 was similarly observed (Fig 7B), showing that 4 peptides, 27, 41, 53, and 81, induced CTLs *in vivo*. No adverse events were induced in mice, including local findings, confirming its safety.

## Discussion

We performed an analysis with 93 peptides, including 4 peptides previously reported, and identified new epitopes that correlated with HBsAg and HBcrAg levels capable of inducing CTLs *in vivo*. The important point that led to the discovery of these epitopes was increasing candidate epitopes by setting a low predicted binding rate in the first computer screening. Inconsistencies have been reported between the binding rate and strength of immune reactions [27], and a low binding rate or no binding was frequently detected in these peptides in the binding assay. In previous studies on HLA-A24-restricted epitopes, peptides with a high binding rate were generally narrowed down in the first step [14, 28]; therefore, setting a low binding rate may have resulted in the discovery of new epitopes. This is important because this method is applicable to other HLA and fields.

Specific cytotoxicity was often not detected in the CTL assay in these peptides. Since these peptides were clearly recognized by CTLs in other experiments and were confirmed to induce the secretion of cytokines, these epitopes were considered to inhibit viral replication or inactivate the virus even though they do not exhibit cytotoxicity. The presence of this type of CTL has been reported [8,29]. One of these peptides was positive in HBV carriers in the IFN-γ-ELISPOT assay. HBV carriers are in the immunosurveillance state, in which intracellular viral replication is inhibited, thereby preventing hepatitis. This finding suggests the presence of an epitope that controls viral replication separately from the route of cell injury by CTLs.

The peptides identified in the present study may be applicable to actual treatments based on comparisons between before and after the administration of NA, as shown in Fig 6. Immune reactions are strengthened after the administration of NA, as described above, suggesting that these peptides serve as epitopes in the bodies of patients. Since many of the peptides tested exhibited this function, they may be recognized as epitopes in the bodies of chronic hepatitis patients, and this immune reaction may be promoted by the administration of peptides.

The measurement of cccDNA in hepatocytes is difficult and labor intensive; however, the correlation between HBcrAg levels and cccDNA has recently been confirmed as a new HBV marker [30]. Therefore, a clearer understanding of the infected state of HBV has been obtained. Since HBV is controlled in patients with low serum HBcrAg levels, these levels will be reduced in patients exhibiting an immune response to a true epitope involved in the control of HBV. In the present study, HBcrAg levels were measured and analyzed in all patients. HBcrAg levels were significantly lower in patients in whom an immune response to peptide 41 was detected, indicating the control of cccDNA. HBsAg levels were also significantly lower in patients in whom an immune reaction to this peptide was detected. It can be understood from the changes in serum HBcrAg, HBsAg and ALT levels that this is the most likely epitope controlling HBV. To the best of our knowledge, no HBV epitope for which an immune reaction correlated with HBsAg and HBcrAg levels has been reported.

Our important discovery is that immune reactions appear to occur with decreases in amino acid mutations in the same HBV genotype C because the induction of an immune reaction to HBV without mutations, and not to HBV with mutations, indicates that the immunogenicity of this region is important.

The involvement of many epitopes, particularly HLA-A2-restricted epitopes, in the elimination of HBV has been reported [11], suggesting that many epitopes are also involved in HLA-A24. A previous study reported that many strongly immunogenic epitopes are present in the core region [31], whereas 1 and 2 of these newly discovered epitopes were present in the surface antigen and polymerase, respectively, and only 1 was present in the core antigen, suggesting that many epitopes in various regions are involved in immunity.

In conclusion, we herein identified new HLA-A24-restricted epitopes that correlated with HBsAg and HBcrAg levels against HBV genotype C. These newly identified epitopes may be useful in the analysis of immune responses against HBV and development of immunotherapy.

## Supporting information

S1 Checklist ARRIVE guidelines checklist. (PDF)

S1 Fig Positive and negative controls of IFN-γ ELISPOT assays. CMVpp65_328_ peptide-specific CTL responses are positive controls. HIVenv584 peptide-specific CTL responses are negative controls. Abbreviations: PBMCs, peripheral blood mononuclear cells; CMV, cytomegalovirus; HIV, human immunodeficiency virus; No., number.
(TIF)

S2 Fig Number of spots of peptides 41, 53, 55, 81, 91, and 92. The number of specific spots in each patient is shown for 6 peptides for which more than four patients were positive. Asterisks indicate more than 100 spots. Peptide sequences are described in S1 Table. Abbreviations: No., number; PBMCs, peripheral blood mononuclear cells.
(TIF)

S3 Fig IFN-γ ELISPOT assays of healthy individuals. IFN-γ ELISPOT assays with 93 peptides were performed for 17 healthy HLA-A24-positive individuals (a-q). Peptides 13, 14, 17, 27, 30, 31, 32, 33, 42, 43, 44, 52, 54, 56, 58, 90, and 91 were positive in only one individual. None of the peptides were positive in 2 or more individuals. Abbreviations: No., number; HIV, human immunodeficiency virus; CMV, cytomegalovirus.
(TIF)

S1 Table Peptide list. (DOCX)
