# Supplementary figures and images for: Immune responses of human T lymphocytes to novel hepatitis B virus-derived peptides
Source: PLoS One. 2018 Jun 1;13(6):e0198264. doi: 10.1371/journal.pone.0198264 (PMC5983448; doi:10.1371/journal.pone.0198264)

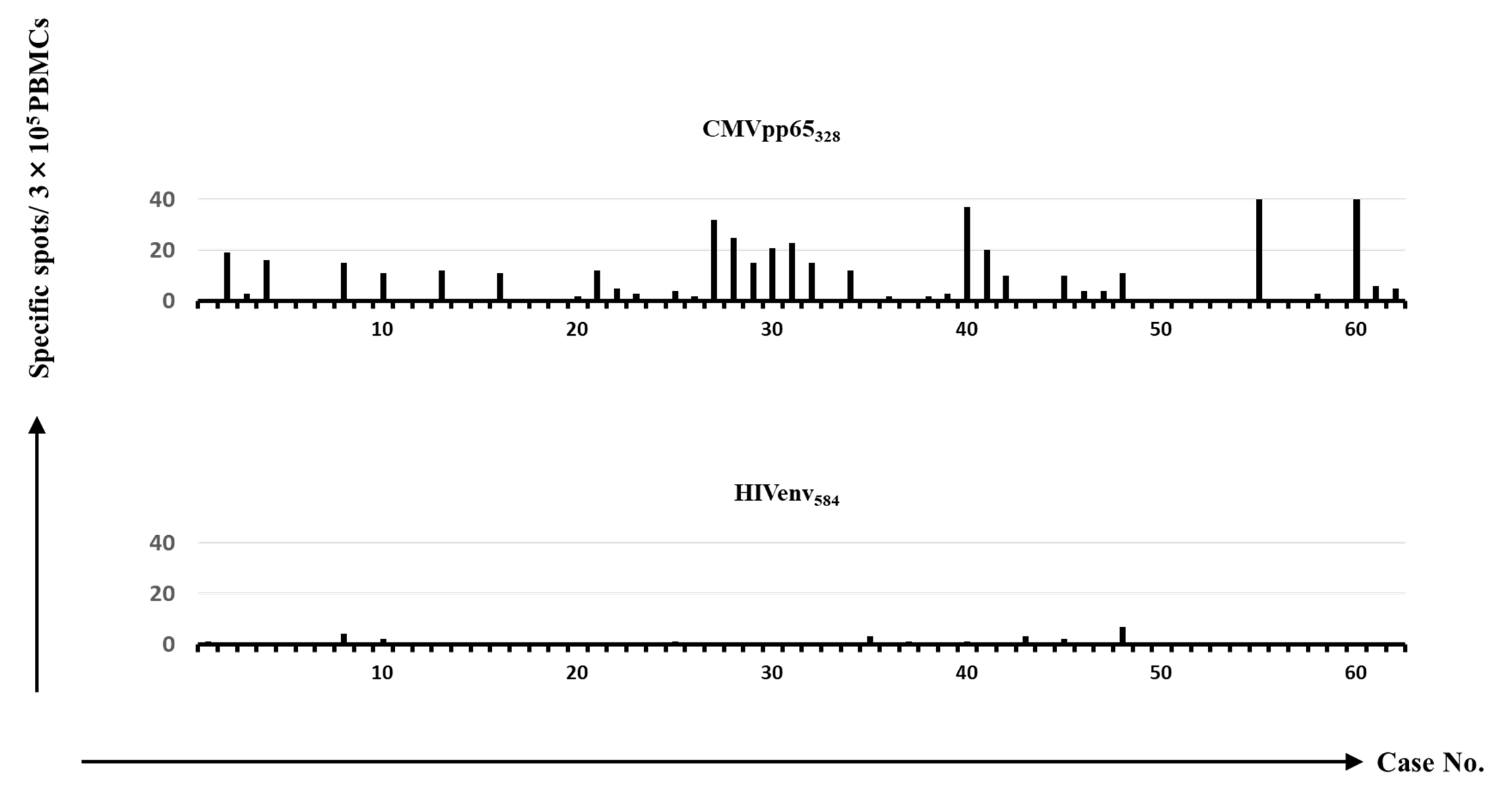

Supplement: S1 Fig — CMVpp65328 peptide-specific CTL responses are positive controls. HIVenv584 peptide-specific CTL responses are negative controls. Abbreviations: PBMCs, peripheral blood mononuclear cells; CMV, cytomegalovirus; HIV, human immunodeficiency virus; No., number. (TIF) [file pone.0198264.s002.tif]

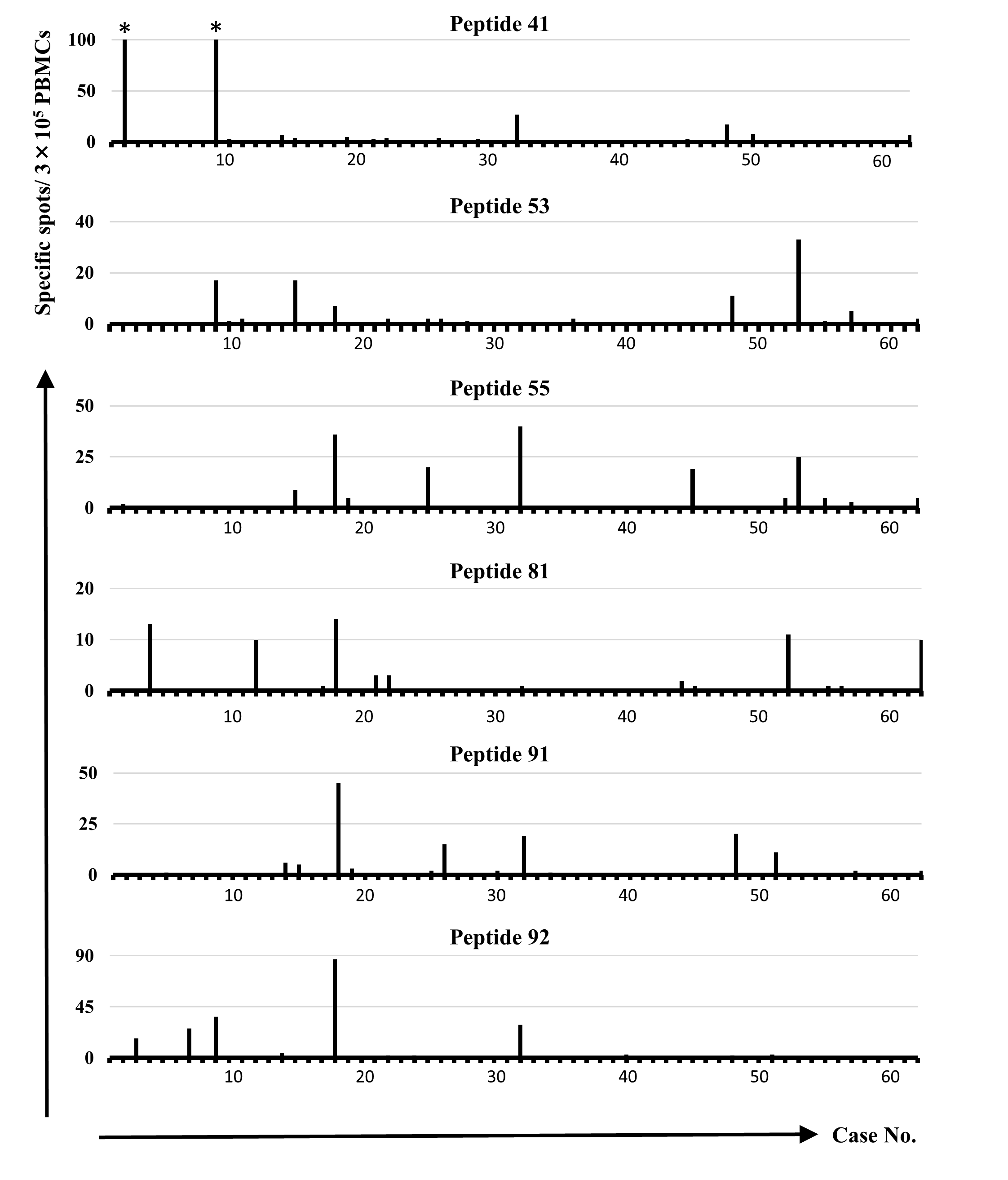

Supplement: S2 Fig — The number of specific spots in each patient is shown for 6 peptides for which more than four patients were positive. Asterisks indicate more than 100 spots. Peptide sequences are described in S1 Table. Abbreviations: No., number; PBMCs, peripheral blood mononuclear cells. (TIF) [file pone.0198264.s003.tif]

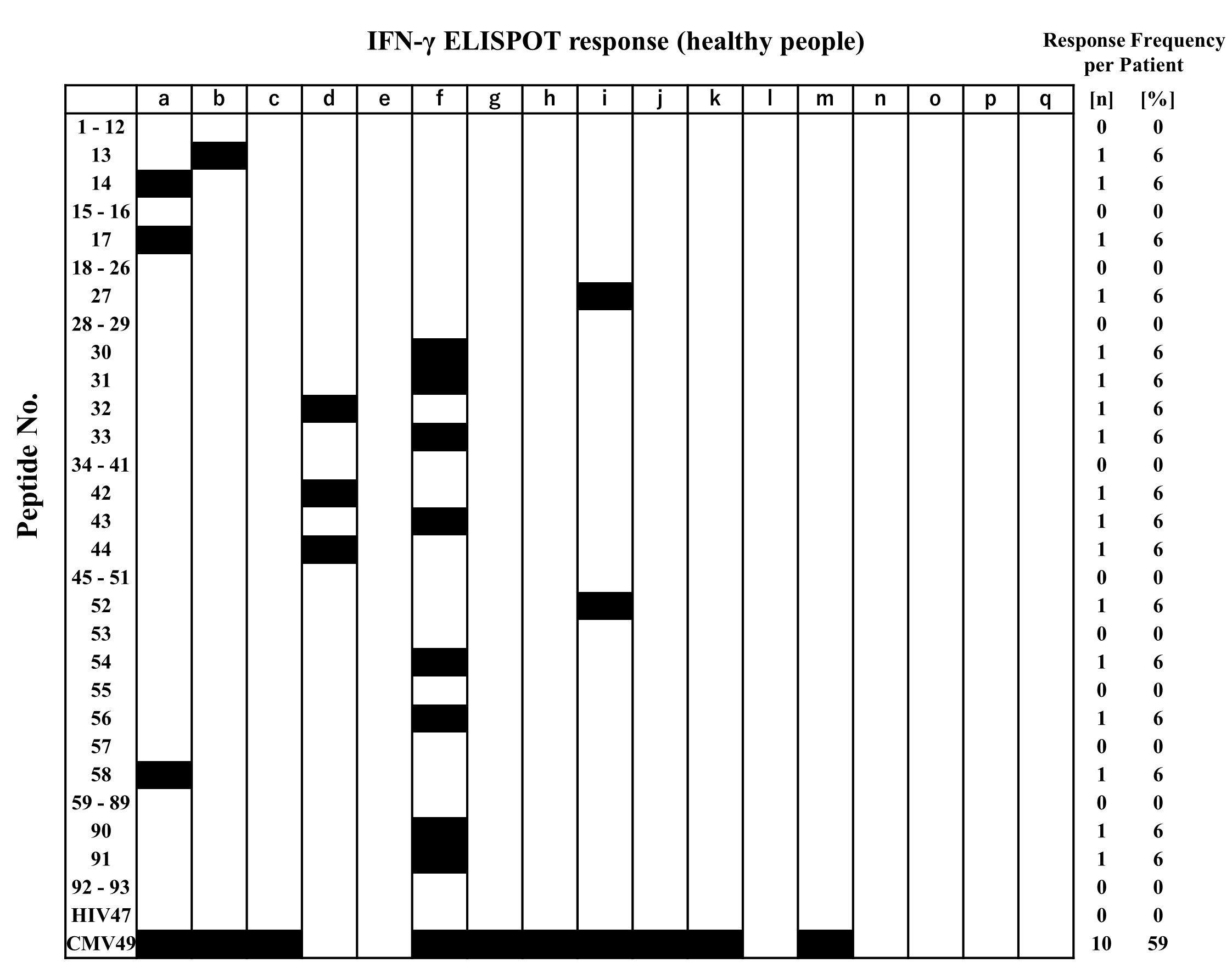

Supplement: S3 Fig — IFN-γ ELISPOT assays with 93 peptides were performed for 17 healthy HLA-A24-positive individuals (a-q). Peptides 13, 14, 17, 27, 30, 31, 32, 33, 42, 43, 44, 52, 54, 56, 58, 90, and 91 were positive in only one individual. None of the peptides were positive in 2 or more individuals. Abbreviations: No., number; HIV, human immunodeficiency virus; CMV, cytomegalovirus. (TIF) [file pone.0198264.s004.tif]
