# Supplementary material for: Immune responses of human T lymphocytes to novel hepatitis B virus-derived peptides
Source: PLoS One. 2018 Jun 1;13(6):e0198264. doi: 10.1371/journal.pone.0198264 (PMC5983448; doi:10.1371/journal.pone.0198264)
Supplement: S1 Table — (DOCX) [file pone.0198264.s005.docx]

| No. | HBV Protein | Amino Acid Position | Amino Acid Sequence | score |  | No. | HBV Protein | Amino Acid Position | Amino Acid Sequence | score |
| --- | --- | --- | --- | --- | --- | --- | --- | --- | --- | --- |
| 1 | Env | 379-387 | LYNILSPFL | 360 |  | 48 | Pol | 4-12 | SYQHFRKLL | 300 |
| 2 | Env | 253-261 | RFIIFLFIL | 60 |  | 49 | Pol | 493-501 | LYKTFGRKL | 220 |
| 3 | Env | 181-189 | GFLGPLLVL | 30 |  | 50 | Pol | 121-129 | KYLPLDKGI | 216 |
| 4 | Env | 192-200 | GFFLLTRIL | 24 |  | 51 | Pol | 140-148 | HYFKTRHYL | 200 |
| 5 | Env | 66-74 | GFTPPHGGL | 24 |  | 52 | Pol | 62-70 | LYSSTVPVF | 100 |
| 6 | Env | 198-206 | RILTIPQSL | 20 |  | 53 | Pol | 776-784 | SFVYVPSAL | 42 |
| 7 | Env | 14-22 | TNLSVPNPL | 10 |  | 54 | Pol | 566-574 | LFTSITNFL | 34 |
| 8 | Env | 276-284 | GMLPVCPLL | 10 |  | 55 | Pol | 484-492 | RNLYVSLLL | 17 |
| 9 | Env | 258-266 | LFILLLCLI | 9 |  | 56 | Pol | 456-464 | RYVARLSST | 15 |
| 10 | Env | 139-147 | LYLPAGGSS | 9 |  | 57 | Pol | 668-676 | TFSPTYKAF | 12 |
| 11 | Env | 375-383 | WGPSLYNIL | 9 |  | 58 | Pol | 91-99 | RCQQYVGPL | 12 |
| 12 | Env | 254-262 | FIIFLFILL | 9 |  | 59 | Pol | 215-233 | RSQLKQSRL | 12 |
| 13 | Env | 273-281 | DYQGMLPVC | 8 |  | 60 | Pol | 558-566 | KSVQHLESL | 12 |
| 14 | Env | 264-272 | CLIFLLVLL | 7 |  | 61 | Pol | 614-622 | KIKQCFRKL | 11 |
| 15 | Env | 261-269 | LLLCLIFLL | 7 |  | 62 | Pol | 674-682 | KAFLCKQYL | 10 |
| 16 | Env | 381-389 | NILSPFLPL | 7 |  | 63 | Pol | 680-688 | QYLNLYPVA | 9 |
| 17 | Env | 188-196 | VLQAGFFLL | 7 |  | 64 | Pol | 650-658 | GYPALMPLY | 9 |
| 18 | Env | 270-278 | VLLDYQGML | 7 |  | 65 | Pol | 486-494 | LYVSLLLLY | 9 |
| 19 | Env | 67-75 | FTPPHGGLL | 7 |  | 66 | Pol | 684-692 | LYPVARQRS | 9 |
| 20 | Env | 104-112 | QPTPISPPL | 7 |  | 67 | Pol | 407-415 | FAVPNLQSL | 9 |
| 21 | Env | 263-271 | LCLIFLLVL | 6 |  | 68 | Pol | 411-419 | NLQSLTNLL | 9 |
| 22 | Env | 187-195 | LVLQAGFFL | 6 |  | 69 | Pol | 210-218 | VGPGVRSQL | 8 |
| 23 | Env | 275-283 | QGMLPVCPL | 6 |  | 70 | Pol | 818-826 | VSPSVPSHL | 8 |
| 24 | Env | 192-200 | IFFCLWVYI | 6 |  | 71 | Pol | 632-640 | VCQRIVGLL | 8 |
| 25 | Env | 260-268 | ILLLCLIFL | 6 |  | 72 | Pol | 146-154 | HYLHTLWKA | 8 |
| 26 | Env | 77-85 | WSPQAQGVL | 6 |  | 73 | Pol | 807-815 | RPTTGRTSL | 8 |
| 27 | Env | 358-366 | VGLSPTVWL | 6 |  | 74 | Pol | 631-639 | KVCQRIVGL | 8 |
| 28 | Env | 373-381 | WYWGPSLYN | 5 |  | 75 | Pol | 268-276 | RASSTSSCL | 8 |
| 29 | Nuc | 116-124 | SYVNVNMGL | 420 |  | 76 | Pol | 143-151 | KTRHYLHTL | 8 |
| 30 | Nuc | 160-168 | AYRPPNAPI | 60 |  | 77 | Pol | 585-593 | KTKRWGYSL | 8 |
| 31 | Nuc | 52-60 | FFPSIRDLL | 42 |  | 78 | Pol | 794-802 | RLGLYRPLL | 8 |
| 32 | Nuc | 51-59 | DFFPSIRDL | 24 |  | 79 | Pol | 495-503 | KTFGRKLHL | 8 |
| 33 | Nuc | 37-45 | EFGASVELL | 20 |  | 80 | Pol | 597-605 | GYVIGCWGT | 8 |
| 34 | Nuc | 122-130 | MGLKIRQLL | 11 |  | 81 | Pol | 94-102 | QYVGPLTVN | 8 |
| 35 | Nuc | 131-139 | WFHISCLTF | 10 |  | 82 | Pol | 387-395 | RLVVDFSQF | 7 |
| 36 | Nuc | 146-154 | EYLVSFGVW | 9 |  | 83 | Pol | 224-232 | GLQPQQGSL | 7 |
| 37 | Nuc | 40-48 | ASVELLSFL | 9 |  | 84 | Pol | 573-581 | FLLSLGIHL | 7 |
| 38 | Nuc | 58-66 | DLLDTASAL | 7 |  | 85 | Pol | 415-423 | LTNLLSSNL | 7 |
| 39 | Nuc | 86-94 | QAILCWGEL | 7 |  | 86 | Pol | 517-525 | MGVGLSPFL | 7 |
| 40 | Nuc | 16-24 | TVQASKLCL | 6 |  | 87 | Pol | 338-346 | CLTHIVNLL | 7 |
| 41 | Nuc | 97-105 | LATWVGSNL | 6 |  | 88 | Pol | 101-109 | VNEKRRLKL | 7 |
| 42 | HBx | 81-89 | TTVNAHQVL | 7 |  | 89 | Pol | 17-25 | EAGPLEEEL | 6 |
| 43 | HBx | 26-34 | RGRPVSGPF | 7 |  | 90 | Env | 89-97 | SWWTSLNFL | * |
| 44 | HBx | 1-9 | MAARVCCQL | 6 |  | 91 | Env | 226-234 | SWLSLLVPF | * |
| 45 | HBx | 93-101 | LYKRTLGLS | 5 |  | 92 | Nuc | 101-110 | LWFHISCLTF | * |
| 46 | Pol | 756-764 | KYTSFPWLL | 400 |  | 93 | Env | 236-245 | RWMCLRRFII | * |
| 47 | Pol | 115-123 | FYPNLTKYL | 360 |  |  |  |  |  |  |

**Supplementary table 1. Peptide list**

Env, Envelope; Nuc, Nucleus; Pol, Polymerase

* peptides which were previously described
